# Association between dietary fat and skin cancer in an Australian population using case-control and cohort study designs

**DOI:** 10.1186/1471-2407-6-141

**Published:** 2006-05-30

**Authors:** Robert H Granger, Leigh Blizzard, Jayne L Fryer, Terence Dwyer

**Affiliations:** 1 Menzies Research Institute, Hobart, Australia; 2 Murdoch Childrens Research Institute, Melbourne, Australia

## Abstract

**Background:**

Human studies of dietary fat as a possible risk factor for cutaneous malignant melanoma (CMM) and non-melanoma skin cancer (NMSC) – principally basal cell carcinoma (BCC) and squamous cell carcinoma (SCC) – have produced inconsistent results. We had the opportunity to examine the association concurrently for all three types of skin cancer in a population-based study in Tasmania, Australia, involving 652 cases of CMM, BCC and SCC and a common set of 471 controls.

**Methods:**

Histopathologically-confirmed cases of CMM, BCC and SCC were ascertained from the Tasmanian Cancer Registry (TCR), and controls were selected at random from the state's electoral roll. We compared subjects categorised by thirds of dietary fat intake score measured by the 'Dobson short fat questionnaire', with logistic regression models that adjusted for age, sex, skin type and usual sun exposure. We then followed all subjects for 56–80 months until 31 August, 2004 for a new NMSC using record linkage with both the TCR and the Births, Deaths, and Marriages registry. Incidence rates were calculated and ratios of rates were estimated using Poisson models.

**Results:**

Relative to subjects in the lowest fat intake category, the odds ratios (OR) comparing cases and controls were OR = 0.76 (95% CI: 0.56–1.03) for medium fat intake, and OR = 0.62 (95% CI: 0.45–0.85) for high fat intake, with a significant (*p *< 0.01) trend of reduced odds ratio with higher category dietary fat intake. Among cases, the incidence rate ratio (IRR) relative to those with lowest fat score was IRR = 0.72 (95% CI: 0.50–1.03) for medium fat intake, and IRR = 0.82 (95% CI: 0.56–1.20) for highest fat intake (linear trend *p *= 0.30).

**Conclusion:**

Using the same dietary instrument with two study designs in the same Caucasian population, we found no evidence that high fat intake increases the risk of developing melanoma or non-melanoma skin cancers. Instead, our results suggest a risk reduction for high fat intake.

## Background

Exposure to ultraviolet radiation is the single most important risk factor in the aetiology of skin cancer [1]. Variations in the incidence of cutaneous malignant melanoma (CMM) between similar populations living at similar latitudes suggest other factors, including diet, may play a role [2]. There is reason to suspect that dietary fat acts as a promoter of carcinogenesis. Experimental studies on mice provide evidence that dietary fat in general, and polyunsaturated fat in particular, may enhance the carcinogenic effects of ultraviolet radiation [3]. A dietary intervention study demonstrated that reduction in fat intake reduces risk of non-melanoma skin cancer (NMSC) [4,5], but the evidence from observational studies [2,6-18] has been mixed.

No study to date has investigated the association between dietary fat intake and risk of CMM, basal cell carcinoma (BCC) and squamous cell carcinoma (SCC) in the same population using the same measure of fat intake. We had the opportunity to do this in a population-based study in Tasmania involving 652 cases of CMM, BCC and SCC and a common set of 471 controls. We then followed these subjects from the case-control study for occurrences of subsequent NMSC to determine whether the results of the case-control study could be confirmed with prospective data.

## Methods

### Study subjects

The methods used in the case-control study are explained in detail elsewhere [19]. In brief, the study population consisted of subjects aged 20–59 years of northern European ancestry who were residents of the state of Tasmania, Australia (latitude 41–44° south), and who had never been previously diagnosed with histologically-confirmed CMM. Eligible cases of CMM were ascertained from notifications to the Tasmanian Cancer Registry between January 1, 1998 and December 31, 1999. Eligible cases of BCC and SCC were a random sample of notifications to the Registry between July 1, 1998 and June 30, 1999. Controls were selected randomly from the state's comprehensive electoral roll, but frequency matched to CMM cases within 5-year age categories. The response proportions were 245 (90.1%) of 272 eligible CMM cases, 224 (88.2%) of 254 eligible BCC cases, 199 (88.1%) of 226 eligible SCC cases, and 490 (80.7%) of 607 persons invited to participate as controls. All subjects in the case-control study provided informed consent prior to participation.

For the cohort study, participants in the case-control study were followed until August 31, 2004 for occurrence of a subsequent NMSC by record-linkage with the Tasmanian Cancer Registry. Deaths in the cohort were identified from the Tasmanian Cancer Registry database, and additionally by linkage with records from the database maintained by the Births, Deaths, and Marriages registry.

### Measures

In the case-control study, participants completed a questionnaire and physical examination. Sun exposure in childhood, teenage years and recent adult life were assessed by questions about the numbers of hours usually spent in the sun during weekends and holidays, along with the frequency of outdoor activities. Natural hair colour at those time points was also reported. Self-assessed skin sensitivity was determined by questions on the subject's tendency to burn and inability to tan. Cutaneous melanin density at the upper inner arm was assessed from measurements of skin reflectance made using a handheld Minolta 508 spectrophotometer. The interviewer also counted naevi on the left arm and back of subjects, and graded each subject's skin colour, eye colour and freckling patterns. These measures are described in detail elsewhere [19].

Dietary fat intake was assessed with the 'Dobson short fat questionnaire' [20], which ranks subjects according to their fat intake. The questionnaire was not designed to assess energy intake. Total fat scores were calculated by summing responses from the 17 questions. Fourteen of those questions had integer values ranging from 0 (never) to 4 (six or more times a week) based on the frequency of consumption of common fat-containing foods. Three items had fewer options: spreading margarine or butter on bread (range 0–3), eating skin of chicken (range 0–2) and removing fat from meat (range 0–2). The questionnaire has been shown to have good criterion validity and reproducibility [20]. Cutaneous melanin density of unexposed skin was measured with a handheld Minolta 508 spectrophotometer [19].

Anthropometric measurements included height and weight from which body mass index was calculated, and waist and hip circumference from which waist-to-hip ratio was calculated. As an indicator of socioeconomic status, each participant's postcode of residence was scored into one of five ordered categories according to the Index of Relative Socioeconomic Disadvantage constructed by the Australian Bureau of Statistics. The Human Research Ethics Committee of the University of Tasmania approved the study protocols.

### Statistical analyses

Analyses were restricted to the 96.1% (642/668) of cases and 96.1% (471/490) of controls with complete dietary fat data. For the case-control study, analysis of variance methods were used to compare mean fat intake scores of cases and controls and Spearman correlations were calculated from the ranks of the data. Logistic regression models were built to compare the odds of high fat scores for cases and controls with adjustment for confounders. For the cohort study, incidence rates were calculated by dividing the number of subjects having a new lesion by the accumulated person-years of follow-up. Person-years were calculated from the initiating date to the first diagnosis of a NMSC lesion (CMM cases) or a new NMSC lesion at least 90 days after the first (NMSC cases), or death, or end of follow-up (August 31, 2004), whichever came first. For those subjects with a prior NMSC (BCC or SCC), a further NMSC of the same type occurring within 90 days was regarded as synchronous and not a new lesion. For controls, the initiating date was the date of interview. Incidence rates for groups of subjects categorised by dietary fat intake were compared using incidence rate ratios (IRR) estimated with Poisson models (log-linear models with Poisson errors and a person-years offset).

In each regression approach, the basic model contained binary (0,1) predictors for categories of fat score other than the lowest. We routinely adjusted for age, the matching factor, and for sex in analyses of data for men and women combined. Trends in the estimated effect of fat score were assessed from the coefficient and standard error of a single linear predictor taking consecutive integer scores (1,2,3) for increasing categories of fat intake. Confounding by factors including age, sex, socioeconomic status, sun exposure and skin phenotype were assessed by the change-in-parameter-estimate approach. Effect modification was assessed from the coefficient and standard error of a product term formed by multiplying a linear term for fat category with a linear term for the potential modifier.

## Results

Mean values of the dietary fat intake scores are shown in Table 1 for cases and controls, together with mean values of a number of body size and shape characteristics. Despite being similar in body size, the controls had higher sex-adjusted mean values (25.9, standard error SE = 0.5) for dietary fat intake than did CMM cases (24.5, SE = 0.5, *p *< 0.01), BCC cases (24.9, SE = 0.5, *p *= 0.07) and SCC cases (24.3, SE = 0.05, *p *< 0.01).

The dietary fat scores were negatively correlated with age for controls (r = -0.14, *p *< 0.01), CMM cases (r = -0.17, *p *< 0.01) and BCC cases (r = -0.29, *p *< 0.01) independently of sex. After additionally adjusting for age, statistically significant associations remained with higher socioeconomic status for BCC cases (r = -0.20, *p *< 0.01) and SCC cases (r = -0.29, *p *< 0.01), with waist-to-hip ratio (r = 0.22, *p *< 0.01) for CMM cases, and with frequency of participation in summer sporting and recreational activities for male controls (r = -0.18, *p *< 0.01) and CMM cases (r = -0.23, *p *= 0.02).

The results of logistic regression analyses of the case-control data are shown in Table 2. For these analyses we divided the fat scores into three categories, each containing about one-third of the data. Uniformly for men and women and for each type of skin cancer, the cases had lower odds of higher fat intake than did controls, though mostly without reaching statistical significance. With all types of skin cancer and both sexes combined, the odds ratios comparing cases and controls were OR = 0.76 (95% CI: 0.52–1.03) for fat score 22–28 points and OR = 0.62 (95% CI: 0.45–0.85) for fat score 29–51 points (linear trend *p *< 0.01). These odds ratio are adjusted for age, sex where appropriate, melanin density in the skin of the upper inner arm, and usual sun exposure as an adult. Higher waist-to-hip ratios were associated with reduced risk of all skin cancers combined (*p *= 0.05), primarily through its association with reduced risk of BCC (*p *< 0.01) for which the age- and sex-adjusted odds ratio was OR = 0.83 (95% CI: 0.69–0.98) for each 0.1 increase in waist-to-hip ratio units. Adjusting for it strengthened the estimated effect of fat intake, albeit marginally. None of the other body size indices were significantly associated with risk, and this was the case also for socioeconomic status. Further analyses revealed no significant effect modification by age, gender, melanin density or sun exposure.

With the objective of confirming these results, we followed this cohort until August 31, 2004 for occurrence of subsequent NMSC. The results in Table 3 show that amongst cases, those with a higher dietary fat category generally had lower subsequent sex-, age- and melanin-adjusted incidence of NMSC than those in a low dietary fat category. The results are not adjusted for sun exposure because we did not have measures of sun exposure during the intervening period. For all types of skin cancer cases combined, the incidence rates relative to those with lowest fat score (1–21 points) were IRR = 0.72(95% CI: 0.50–1.03) for subjects with fat score 22–28 points and IRR = 0.82(95% CI: 0.56–1.20) for subjects with fat score 29–51 points (linear trend *p *= 0.30). These incidence rate ratios are adjusted for age, sex and melanin density in the skin of the upper inner arm. Excluding subjects who had a NMSC prior to the study period made little difference to these results. The corresponding results for controls were IRR = 5.5 (95% CI: 0.7–45.1) for those with fat score 22–28 points, and IRR = 2.9 (95% CI: 0.3–26.7) for those with fat score 29–51 points. The confidence intervals are wide because only 12 controls had a NMSC during follow-up.

## Discussion

To our knowledge, this is the first study to investigate associations of fat intake with CMM, BCC and SCC using the same measure of fat intake. The results from our case-control study do not suggest that the risk of CMM, BCC and SCC is increased with higher dietary fat intake. In fact, our odds ratio estimates suggest that risk is reduced. The results from the follow-up of cases also suggest reduced risk for higher fat intake.

Our results do not support those of an intervention study which examined the effect of a low-fat diet on incidence of NMSC [4]. During the final 8 months of the 2-year trial, during which just 7 patients experienced lesions, one of the 57 patients in the intervention group and 6 of the 58 patients in the control group experienced a lesion. Our results are consistent with the findings of a cohort study of BCC in which risk was found to be inversely associated with intake of total and monounsaturated fat intake [15]. Findings from a different cohort study [11] and nested case-control study of BCC subjects [7], though, showed no associations for energy-adjusted fat intake. Nor was a clear dose-related association in either direction observed for CMM in a large cohort in Norway [2].

Results of case-control studies have been mixed. In a small Australian study, 41 women with CMM had significantly lower odds of being in a higher fat intake category than did controls (n = 271) for total, mono- and polyunsaturated fat intake [6]. Among the many and inconsistent results of the case-control study by Hakim [10], SCC cases had lower odds of higher n-3 fatty acid intakes (linear trend p = 0.06). Other case-control studies found no significant associations with fat intake [7-9,11,13,16].

In a recent review of all human studies investigating dietary factors in NMSC, McNaughton [21] identified several limitations of the observational studies. These limitations may account in part or full for the inconsistencies in study findings. They include reliance on self-reports of BCC outcomes in cohort studies [11-15], low response fractions [14], hospital-based selection of controls [9] and limited dietary assessment [9]. Our study avoided some of these limitations, because disease outcomes were verified by histopathological assessment, and there was population-based ascertainment of cases and selection of controls. Together with high response fractions, the possibility of selection bias was restricted.

On the other hand, our study is limited by the imprecision of the dietary fat assessment instrument, as measurement error of diet has already been shown to obscure the relationship between dietary fat and breast cancer [22]. We were unable to quantify the intake of fat and its various constituents (total, saturated, mono- and polyunsaturated) using the Dobson 'short fat questionnaire' instrument. The criterion validity of the instrument for total fat and saturated fat as a percentage of energy intake were assessed by comparing it with the Commonwealth Scientific and Industrial Research Organization's food frequency questionnaire (r = 0.67; r = 0.55 respectively) [20]. Reproducibility of scores seven to nine months after the initial assessment achieved a respectable correlation of r = 0.85. In our sample, the measurements appeared to have construct validity in that higher intakes were associated with lower socioeconomic status, greater body size, and reduced sports participation. None of these factors appeared to explain the associations found, though the possibility of residual confounding cannot be dismissed.

A further limitation of our analyses was the inability to adjust for total energy consumption, which was not measured by our dietary questionnaire. We are unable to demonstrate that the effect of fat intake is independent of total caloric intake [23]. Further, we cannot dismiss the possibility that the higher fat intake of those subjects at lowest risk of skin cancer constituted a smaller proportion of their total energy intake, although their similar body sizes mitigates against this possibility. It is also reassuring to note that total energy intake was not observed to predict risk of NMSC in previously-cited studies [5,7,10,11,15,24], although we are not aware of any study which has directly tested this relationship.

Whilst a single dietary intervention study has been conducted [4,5,24] that included rigorous monitoring of outcomes and diet, inferences were drawn from a relatively small number of events using questionable analytical methods [15]. McNaughton [21] points out that the fat reduction was accompanied by increased intake of vitamin C, β-carotene and fibre, though there was no difference between experimental and control groups with respect to total calories consumed. This suggests an increase in consumption of fruit and vegetables that are recognised as playing a protective role in many types of cancers, leaving open the possibility that the risk reduction found in that study is attributable, in part, to dietary factors other than reduced fat intake.

## Conclusion

In summary, using the same dietary instrument in a study with population-based ascertainment of cases recruited with high response rates, we found no evidence that high fat intake increases the risk of development of melanoma and non-melanoma skin cancers. Instead, our results suggest a risk reduction for high fat intake.

## Competing interests

The author(s) declare that they have no competing interests.

## Authors' contributions

RG: data analysis, writing of document, responsible for application to institutional review board, ensured that data linkage with cancer registry took place

LB: data analysis and added substantially to writing of final document, data interpretation

JF: data analysis

TD: guided study concept and design, reviewed final submission and helped with data interpretation

All authors read and approved the final manuscript.

## Pre-publication history

The pre-publication history for this paper can be accessed here:



## Figures and Tables

**Table 1:** Characteristics of subjects who completed the dietary fat questionnaire in the case-control study of cutaneous malignant melanoma (CMM) and basal cell (BCC) and squamous cell (SCC) carcinoma of the skin.

Subject characteristic	Controls	CMM	BCC	SCC
				
	Mean (SD)†	Mean (SD)†	Mean (SD)†	Mean (SD)†
*Men*	(n = 234)	(n = 105)	(n = 114)	(n = 100)
Age (years)	44.7 (9.8)	46.1 (10.0)	45.4 (8.6)	48.7 (6.9)
Fat score (points) *	27.4 (7.1)	26.2 (7.5)	25.9 (8.3)	26.5 (7.1)
Weight (kgs)	83.1 (13.9)	86.2 (13.1)	82.4 (13.1)	83.7 (12.6)
Body mass index (kg/m^2^)	27.1 (4.1)	27.9 (4.0)	26.7 (3.9)	27.3 (3.2)
Waist (cms)	96.5 (11.3)	99.1 (9.9)	95.3 (10.8)	97.2 (9.8)
Waist-to-hip ratio	0.97 (0.06)	0.98 (0.05)	0.96 (0.06)	0.98 (0.05)
Sport participation (times)‡	4.6 (8.9)	5.1 (9.6)	5.8 (11.7)	5.0 (10.5)
Socioeconomic status §	3.0 (1.4)	3.1 (1.5)	3.3 (1.4)	2.8 (1.5)
				
*Women*	(n = 234)	(n = 150)	(n = 119)	(n = 103)
Age (years)	45.3 (9.7)	44.0 (10.0)	45.2 (9.4)	51.7 (5.3)
Fat score (points) *	24.3 (6.9)	22.4 (7.5)	23.2 (7.4)	22.2 (8.0)
Weight (kgs)	70.1 (14.2)	70.4 (13.1)	69.8 (16.1)	72.8 (17.8)
Body mass index (kg/m^2^)	26.8 (5.3)	26.2 (4.0)	26.1 (5.8)	27.7 (6.5)
Waist (cms)	96.3 (13.9)	94.6 (12.6)	94.5 (14.8)	99.5 (15.7)
Waist-to-hip ratio	0.94 (0.06)	0.93 (0.06)	0.93 (0.06)	0.95 (0.06)
Sport participation (times)‡	1.7 (5.3)	2.5 (7.8)	2.9 (8.5)	4.5 (10.5)
Socioeconomic status §	3.0 (1.4)	3.2 (1.5)	3.2 (1.6)	3.2 (1.6)

NOTES

* Frequency of consumption of 17 foods high in fat.

† Mean (standard deviation).

‡ Self-reported frequency of participation in 7 sun-based sporting activities (tennis, softball, baseball, bowls, golf, athletics and cricket) each summer, on average, during the last 5 years.

§ Index of Relative Socioeconomic Disadvantage compiled by the Australian Bureau of Statistics and based on postcode of residence, with five ordered categories (fifths of the distribution) scored with consecutive integer values from 1 (highest disadvantage) to 5 (lowest disadvantage).

**Table 2:** Odds ratio estimates of relative risk of melanoma (CMM) and of basal cell (BCC) and squamous cell (SCC) carcinoma of the skin, for dietary fat intake.

Fat score*	Controls	CMM		BCC		SCC	
				
	N†	N†	OR(95%CI)‡	N†	OR(95%CI)‡	N†	OR(95%CI)‡
*Men*							
1–21 points	45	24	1.00	30	1.00	22	1.00
22–28 points	79	39	1.14(0.59–2.21)	35	0.70(0.37–1.34)	34	1.02(0.52–2.01)
29–51 points	102	36	0.73(0.38–1.41)	41	0.53(0.28–1.00)	40	0.91(0.48–1.76)
Trend			*p *= 0.26		*p *= 0.05		*p *= 0.74
							
*Women*							
1–21 points	81	59	1.00	50	1.00	46	1.00
22–28 points	95	55	0.78(0.48–1.26)	37	0.60(0.35–1.03)	33	0.85(0.47–1.52)
29–51 points	69	29	0.55(0.32–0.97)	26	0.62(0.34–1.12)	17	0.50(0.25–0.99)
Trend			*p *= 0.04		*p *= 0.09		*p *= 0.06
							
*Both sexes*							
1–21 points	126	83	1.00	80	1.00	68	1.00
22–28 points	174	94	0.90(0.61–1.33)	72	0.67(0.44–1.00)	67	0.85(0.55–1.32)
29–51 points	171	65	0.61(0.40–0.92)	67	0.60(0.39–0.91)	57	0.70(0.45–1.10)
Trend			*p *= 0.02		*p *= 0.02		*p *= 0.12

NOTES

* Based on frequency of consumption of 17 foods high in fat.

† Number of subjects.

‡ Odds ratio (95% confidence interval) adjusted for age, density of cutaneous melanin at the upper inner arm, and reported usual sun exposure as an adult.

**Table 3:** Incidence rate ratios of non-melanoma skin cancer during follow-up to 2004, for subjects with a cutaneous malignant melanoma (CMM), basal cell carcinoma (BCC) or squamous cell carcinoma (SCC) of the skin during 1998–99 classified by their self-reported dietary fat intake at that time.

Fat score*	Events†	PY‡	Rate§	IRR (95%CI)¶
*Both sexes*				
	CMM			
	
1–21 points	12	426.1	28.2	1.00
22–28 points	11	495.5	22.2	0.72(0.31–1.68)
29–51 points	9	327.6	27.5	0.82(0.32–2.05)
Trend				p = 0.64
	BCC			
	
1–21 points	38	311.5	122.0	1.00
22–28 points	28	303.9	92.1	0.67(0.39–1.14)
29–51 points	31	270.4	114.7	0.83(0.48–1.42)
Trend				p = 0.51
	SCC			
	
1–21 points	19	325.2	58.4	1.00
22–28 points	20	317.9	62.9	0.79(0.41–1.55)
29–51 points	17	273.5	62.2	0.83(0.42–1.67)
Trend				p = 0.61

NOTES

* Based on frequency of consumption of 17 foods high in fat.

† Number of new BCC or SCC lesions during follow-up.

‡ Person-years of observation from date of diagnosis in 1998–99 to end of follow-up on 31 August, 2004.

§ Incidence per 1,000 person-years.

¶ Incidence rate ratio (95% confidence interval) adjusted for sex, age and density of cutaneous melanin at the upper inner arm.
